# The effects of a rhythm and music-based therapy program and therapeutic riding in late recovery phase following stroke: a study protocol for a three-armed randomized controlled trial

**DOI:** 10.1186/1471-2377-12-141

**Published:** 2012-11-21

**Authors:** Lina Bunketorp Käll, Åsa Lundgren-Nilsson, Christian Blomstrand, Marcela Pekna, Milos Pekny, Michael Nilsson

**Affiliations:** 1 Center for Brain Repair and Rehabilitation, Department of Clinical Neuroscience and Rehabilitation, Institute of Neuroscience and Physiology, Sahlgrenska Academy at University of Gothenburg, Gothenburg, Sweden; 2 Department of Clinical Neuroscience and Rehabilitation, Institute of Neuroscience and Physiology, Sahlgrenska Academy at University of Gothenburg, Gothenburg, Sweden; 3 Hunter Medical Research Institute, University of Newcastle, Newcastle, Australia

## Abstract

**Background:**

Stroke represents one of the most costly and long-term disabling conditions in adulthood worldwide and there is a need to determine the effectiveness of rehabilitation programs in the late phase after stroke. Limited scientific support exists for training incorporating rhythm and music as well as therapeutic riding and well-designed trials to determine the effectiveness of these treatment modalities are warranted.

**Methods/Design:**

A single blinded three-armed randomized controlled trial is described with the aim to evaluate whether it is possible to improve the overall health status and functioning of individuals in the late phase of stroke (1-5 years after stroke) through a rhythm and music-based therapy program or therapeutic riding. About 120 individuals will be consecutively and randomly allocated to one of three groups: (T1) rhythm and music-based therapy program; (T2) therapeutic riding; or (T3) control group receiving the T1 training program a year later. Evaluation is conducted prior to and after the 12-week long intervention as well as three and six months later. The evaluation comprises a comprehensive functional and cognitive assessment (both qualitative and quantitative), and questionnaires. Based on the International classification of functioning, disability, and health (ICF), the outcome measures are classified into six comprehensive domains, with participation as the primary outcome measure assessed by the Stroke Impact Scale (SIS, version 2.0.). The secondary outcome measures are grouped within the following domains: body function, activity, environmental factors and personal factors. Life satisfaction and health related quality of life constitute an additional domain.

**Current status:**

A total of 84 participants were randomised and have completed the intervention. Recruitment proceeds and follow-up is on-going, trial results are expected in early 2014.

**Discussion:**

This study will ascertain whether any of the two intervention programs can improve overall health status and functioning in the late phase of stroke. A positive outcome would increase the scientific basis for the use of such interventions in the late phase after stroke.

**Trial registration:**

Clinical Trials.gov Identifier: NCT01372059

## Background

Stroke is a multifaceted and complex condition. It is the second leading cause of death and a major cause of long-term disability worldwide [1,2], constituting an enormous cost to the society. Stroke often results in physical [3-6], cognitive [3,6], psychological [7-15], and social impairment [13,16,17] and the personal burden of being a stroke survivor is often devastating and has major consequences for the patient’s quality of life [18].

Previous research in the field has primarily focused on treatment in the acute phase and rehabilitation during the first year after stroke. Experimental animal research and neuroimaging studies have provided insight into various aspects of neural plasticity, i.e. possible mechanisms of structural and functional neural reorganisation in the brain following injury [19-21]. The understanding of the brain’s plastic properties has lead to the emergence of new approaches in stroke rehabilitation [22]. A number of animal studies demonstrated that various forms of multimodal (multisensory) stimulation or an enriched environment, facilitate multiple processes in the brain and are associated with improved functional outcome and neural plasticity [23-33].

There is a growing interest in using music and rhythm as a stimulus for neurotherapy [34,35]. Music therapy is the clinical and evidence-based use of music interventions to accomplish individualized goals within a therapeutic relationship by professionals who have completed an approved music therapy program [36]. The new approach to clinical practice and research, known as Neurologic Music Therapy (NMT) [37,38] is based on a neuroscience model of music perception and production, and the influence of music on functional changes in the brain and behaviour functions [39]. Using standardized treatment protocols, NMT is a therapeutic application of music to cognitive, sensory, and motor dysfunctions due to neurologic disease of the nervous system [40]. Music Supported Therapy and Melodic Intonation Therapy were proposed to induce plastic changes in the brain in terms of functional connectivity and neural reorganization in the sensorimotor cortex [41-43], as well as in white matter tracts [44].

Schneider et al. [45,46] showed that Music Auditory Stimulation leads to improvements in speed, precision and smoothness of movements in fine as well as gross motor skills in stroke patients. Further, music therapy has a positive effect on mood in patients with stroke [47-49]. Rhythmic Auditory Stimulation can enhance gait ability [50-52], flexibility [49], as well as functional motor performance of the paretic upper extremity [53]. Despite the growing body of scientific evidence in favour of the use of music therapy in neurorehabilitation, there is a need for better understanding of the impact of the therapy programs incorporating music and rhythm.

Therapeutic riding (TR), also named Equine-Assisted Therapy and Adaptive Riding uses equine-assisted activities for the purpose of contributing positively to cognitive, physical, emotional and social well-being of people with disabilities [54]. In Sweden, TR is used both in neurologically disabled adults and children. TR incorporates mounted activities and exercises, and the patient actively interacts and influences the horse. The movement of the horse affects the patient both physically and psychologically. In contrast to hippotherapy [55], defined as a physical, occupational, and speech-language therapy treatment strategy that utilizes equine movement as part of an integrated intervention program to achieve functional outcomes, TR teaches specific skills and techniques associated with riding a horse. The primary focus is on developing balance, body awareness and muscle tone in the rider by responding and interacting passively to the horse’s multidimensional movement. Given the varying degree of impairment present among stroke survivors, TR has some risks, as do other animal-assisted therapies. However, in accredited centres these risks are minimal and the benefits are likely to outweigh them.

There is limited scientific evidence suggesting that TR is effective. Previous studies indicate that TR and hippotherapy are beneficial for improving postural control in children with cerebral palsy [56-58], patients with multiple sclerosis [59,60], and spinal cord injuries [61]. A positive effect of hippotherapy associated with conventional physical therapy was shown on gait training in post-stroke hemiparetic individuals [62]. The ultimate goals of rehabilitation interventions for stroke survivors as well as the measurement level selection can be guided by the International classification of functioning, disability, and health (ICF) [63-65], namely health-related domains that are assessed from the body, individual and societal perspectives: the domain body functions and structure and the domains of activity and participation. Functioning is an umbrella term encompassing all of the domains mentioned above [66], and participation is considered to be a critically important outcome indicator in the rehabilitation context [67].

Based on the fundamental principles of brain plasticity, we developed rhythm and music-based therapy and TR protocols and designed a three-armed randomized controlled trial to evaluate whether improvement in overall health status and functioning can be achieved in the late post stroke phase by using these multimodal therapy programs. We selected measures most likely to capture change in the targeted aspects of interventions, linked them to the ICF model, and hypothesize that both methods have primary effects on the individuals’ level of participation. To identify potential biomarkers predictive of outcome, blood samples taken at several time points during the study will be analysed. Such biomarkers might ultimately aid to individually tailor neurorehabilitation programs.

### Study aims and objectives

The aim of this study is to investigate whether overall health status and functioning can be improved in community-dwelling individuals in the late phase of stroke through rhythm and music-based therapy or TR. The primary aim is to investigate whether improvement in terms of participation is attained after completion of these therapy programs. Secondary aims are to investigate whether these two interventions have a positive effect on body function, activities, environmental and personal factors, as well as life satisfaction and health related quality of life in the late phase of stroke. In addition, using interviews and focus groups as a qualitative research approach, this study aims at understanding which factors related to the therapies delivered can positively affect stroke survivors’ lives. We also aim at the identification of potential biomarkers in plasma predictive of outcome.

## Methods

### Study design

**Figure 1:**
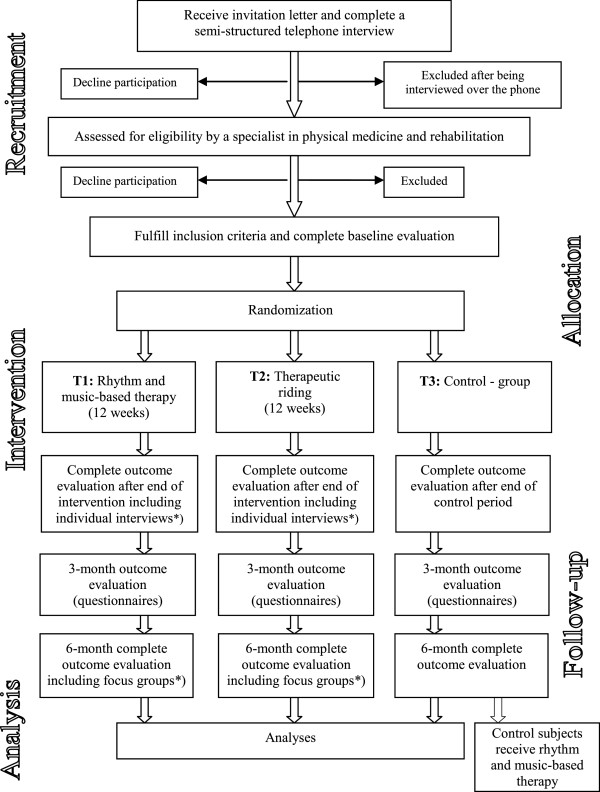
**Flowchart illustrating trial design.** *) Including selected participants.

A single blinded three-armed randomized controlled trial is designed with the aim to evaluate whether it is possible to improve overall health status and functioning of individuals in the late phase of stroke (1-5 years after stroke) through a rhythm and music-based therapy program or TR (Trial registration: Clinical Trials.gov Identifier: NCT01372059). The term *single blinded* refers to the evaluators in the trial being unaware of the nature of the treatment the participants are receiving. About 120 individuals will be consecutively and randomly allocated to one of three groups: (T1) rhythm and music-based therapy program; (T2) TR; or (T3) control group that receives the T1 therapy a year later. Evaluation is conducted prior to and after the 12-week long intervention, and three and six months after completed intervention. The evaluation comprises comprehensive functional and cognitive assessment (both qualitative and quantitative), and questionnaires. Blood samples will be collected pre-, post intervention and at the three and six month follow-up with the aim of identifying potential biomarkers predictive of outcome. Based on the International classification of functioning, disability, and health (ICF), the outcome measures are classified into six comprehensive domains, with participation as the primary outcome measure assessed by the Stroke impact scale (SIS, version 2.0.). The secondary outcome measures are grouped within the following domains: body functions, activity, environmental factors and personal factors. Life satisfaction and health related quality of life constitute an additional domain. The trial design is illustrated in Figure 1. Ethical approval was granted by the Regional Ethical Review Board in Gothenburg (Ref number: 698-09) and the study is conducted in accordance with relevant ethical guidelines.

### Recruitment and selection of participants

The participants are recruited from a comprehensive hospital-based register covering all patients who were treated for ischemic or hemorrhagic stroke at Sahlgrenska University Hospital, Gothenburg, Sweden. The search criterion is limited to individuals who suffered from stroke 1 to 5 years prior to their potential inclusion in the clinical trial. By searching through hospital files, an authorized research coordinator selects persons potentially eligible for the trial. These individuals are then contacted through a letter containing information about the research trial and its implementation. A few days later, each individual receives a follow-up telephone call from the research coordinator. The research coordinator informs the individuals about the study, including requirements, nature and potential benefits of the two therapy programs. Consenting interested subjects are screened by a telephone interview in order to ascertain eligibility for the trial. Further, we record parameters of general health (past and current), medication, physical and cognitive function, stroke-related disability, rehabilitation history, history of previous stroke or other diseases or injuries. Also, information about social relationship, work situation, living arrangements, and the need for transportation service for disabled is obtained.

Individuals who have met the study criteria are invited for a personal appointment with a specialist in rehabilitation medicine for a more detailed screening assessment and interview prior to inclusion. A prior history of stroke is accepted if the previous stroke event affected the same hemisphere as the most recent stroke. Inclusion in the trial requires acceptance of allocation and compliance to either of the three treatment arms, even though allocation to group 3 means that treatment would be delayed by a year. Participants are informed that they can withdraw from the study at any time. After the screening procedure, eligible individuals are included in the trial. Once included, all participants sign a written informed consent form. Selection criteria for trial eligibility are presented below:

Inclusion criteria

– Aged 50 − 75 years

– Disability grade 2 or 3 on MRS*)

– Being in the late-phase of stroke (1 − 5 years after an ischemic or hemorrhagic stroke with initial presence of hemispheric impact/symptoms)

– Subarachnoid hemmorhage with initial presence of hemispheric impact/symptoms

– Ability to understand written and oral information and instructions in Swedish

– Having an own housing

– Ability to travel to the place of intervention and evaluation

– No need for personal assistance in activities of daily living while participating in the treatment (going to the toilet, transport/transportation services for disabled, walking)

– Accepting allocation to either of the three groups which might mean accepting staying without any of the treatment procedures for one year

Exclusion criteria

– Disability rated ^<^ 2 or ^>^ 3 on MRS*)

– An ischemic or hemorrhagic stroke or subarachnoid hemmorhage without hemispheric impact/symptoms

– Pronounced fear of horses or allergy constituting a risk for the patients to participate in the therapeutic riding

– Heart conditions that constitutes a risk for the individual to participate in the interventions

– Non-controlled epileptic seizures constituting a risk for the patients to participate in the interventions

– Lack of cognitive and/or verbal ability or visual impairment that makes it difficult for the individual to understand instructions and/or evaluation

– Total paralysis of the affected arm

– Injury or disease that makes the individual not suitable for the trial

– Weight ^>^ 97 kg (to optimize safe horseback riding)

– Having more than a half-time employment

– Injury, disease or addiction that make the individual not suitable for the trial

– Participation in RGRM or therapeutic riding during the year prior to inclusion

– Having an additional stroke within the past year (TIA is however accepted)

– Lack of willingness to participate in any of the treatment methods

– Living ^>^ 80 km from Gothenburg

– Dependent on transportation services for disabled across the community border which is not allowed according to the regulations

*) Modified Rankin Scale: An ordinal disability rating scale ranging from zero to 6 (0 = no symptoms). MRS grade 1: No significant disability despite symptoms; able to carry out all usual duties and activities; MRS grade 2 = *Slight disability*: unable to carry out all previous activities but able to look after own affairs without assistance; MRS grade 3 = *Moderate disability*: requiring some help, but able to walk without assistance; MRS grade 4: *Moderately severe disability*: unable to walk without assistance and unable to attend to own bodily needs without assistance; MRS grade 5: *Severe disability*: bedridden, incontinent and requiring constant nursing care and attention; MRS grade 6: Dead.

### Randomization, allocation concealment and blinding

As gender and laterality might influence outcome in clinical studies [68], the randomization is stratified with respect to gender and hemispheric location of the stroke (right or left hemisphere). Prior to inclusion of participants, a statistician performed randomization using random permuted blocks for each of the 2 × 2 strata. Until the completion of the last long-term follow-up, only the project leader and two persons responsible for the interviews and focus groups will have access to the information on group allocation. Due to the nature of the therapy programs, blinding of the participants and treating therapists is not possible. However, all the independent evaluators are blinded with respect to group allocation, and the participants are not informed of primary outcome measure or the study hypothesis. To maintain group allocation confidential, participants are requested prior to each assessment phase to not reveal allocation or therapy content to the evaluators. Participants scheduled for qualitative studies are told that they must not talk to the evaluators about participation in interviews and focus groups. Furthermore, the interviews and focus groups are performed in a way that does not reveal participants’ allocation.

### Sample size

The calculation of sample size is based on a clinically relevant difference to be detected across the two main arms of the trial with an alpha level of 5% and a power goal of 80%. A Chi-square test was used for statistical calculations in Nquery 6.0. The required sample size was determined on the basis of one of the items included in the primary stroke-specific, comprehensive, health status outcome measure - Stroke Impact Scale (SIS). SIS measures the aspects of stroke recovery which were found to be important to patients and caregivers as well as stroke experts. The questions of the SIS are broken down into eight domains: strength, hand function, mobility, activities of daily living, emotion, memory, communication, and social participation. The first four of these domains may be combined into one physical domain, but in order to more clearly track changes based on the patient’s particular set of symptoms, the other four items are scored separately. One additional item is included in the SIS to assess the subject’s overall perception of recovery. This item 9 – “Stroke recovery” is presented in the form of a visual analogue scale from 0 to 100 where 0 indicates “no recovery” and 100 indicates “full recovery”.

Based on a previous study indicating that the item of stroke recovery in SIS is a good measure of individual patient’s changes due to rehabilitation [69], and the heterogenic characteristic of the study population, the required sample size was determined based on this specific item. The minimal important difference (MID) was determined a priori. On the basis of previous estimates [70], MID was set at 10 points of the total range of the scale in item 9. Thus, a change of 10 points is considered to represent a meaningful difference. An absolute difference between the two groups of 30% is defined to be a clinically meaningful difference with regard to the item stroke recovery in SIS. Based on this estimate, at least 41 patients would be required in each of the three groups for the results to satisfy the power criteria of 80%.

### Statistical analysis

Outcome variables will be analyzed according to an intention-to-treat model including all randomized patients for whom a baseline value exists in the primary outcome variable. Those who withdraw will be assigned an outcome score identical to their baseline score i.e. no change. In addition, the per-protocol analysis will be restricted to the participants who complete the treatment program and remain in the group to which they were randomly assigned, and have available data on the primary and secondary outcome variables. Patients will be excluded from the per-protocol analysis if they withdrew during the intervention phase or undergo a co-intervention during the three-month intervention phase. Reasons for early withdrawal will be noted.

Baseline and demographic characteristics will be summarized using descriptive statistics. Statistical difference between treatments with respect to the item “stroke recovery” is to be tested using the chi-square test (Mantel Haenzel corrected for gender and hemispheric location) by dichotomizing data into categories *improved* or *unchanged/deteriorated* with respect to change from baseline to follow-up, where improvement/clinically meaningful change is defined as any increase equivalent to 10 points of the total range of the scale. Un-ordered categorical data will be analyzed using the Mantel Haenzel chi-square approach. Analysis of covariance (ANCOVA) will be used to determine whether there are any differences between the control and intervention groups in post-intervention evaluation scores for continuous data, with baseline scores used as covariates in the analysis, and gender, hemispheric location and intervention as fixed factors. Qualitative data from the interviews and focus groups will be analyzed using content analysis as described by Malterud [71]. Unsupervised algorithms will be used to analyze the plasma profiles in search for predictive biomarkers and biomarker patterns predictive of therapeutic success. For the primary and secondary analyses, missing data will be replaced using a conservative method, i.e. last observation carried forward (LOCF). All tests will be two-sided and with p<0.05 as a level of significance.

### Interventions

According to consort and trend guidelines for intervention reporting [72], the rationale for the intervention selection as well as the specification of how the qualities and delivery of the therapeutic modalities are expected to impact targeted outcomes are presented below. The therapeutic modalities used share many therapeutic goals and similarities in the way they combine information from different sensory modalities aiming at enhancing various brain functions. However, due to the way the methods are organized they differ somewhat in terms of dosage.

#### Rhythm and music-based therapy program

**Figure 2:**
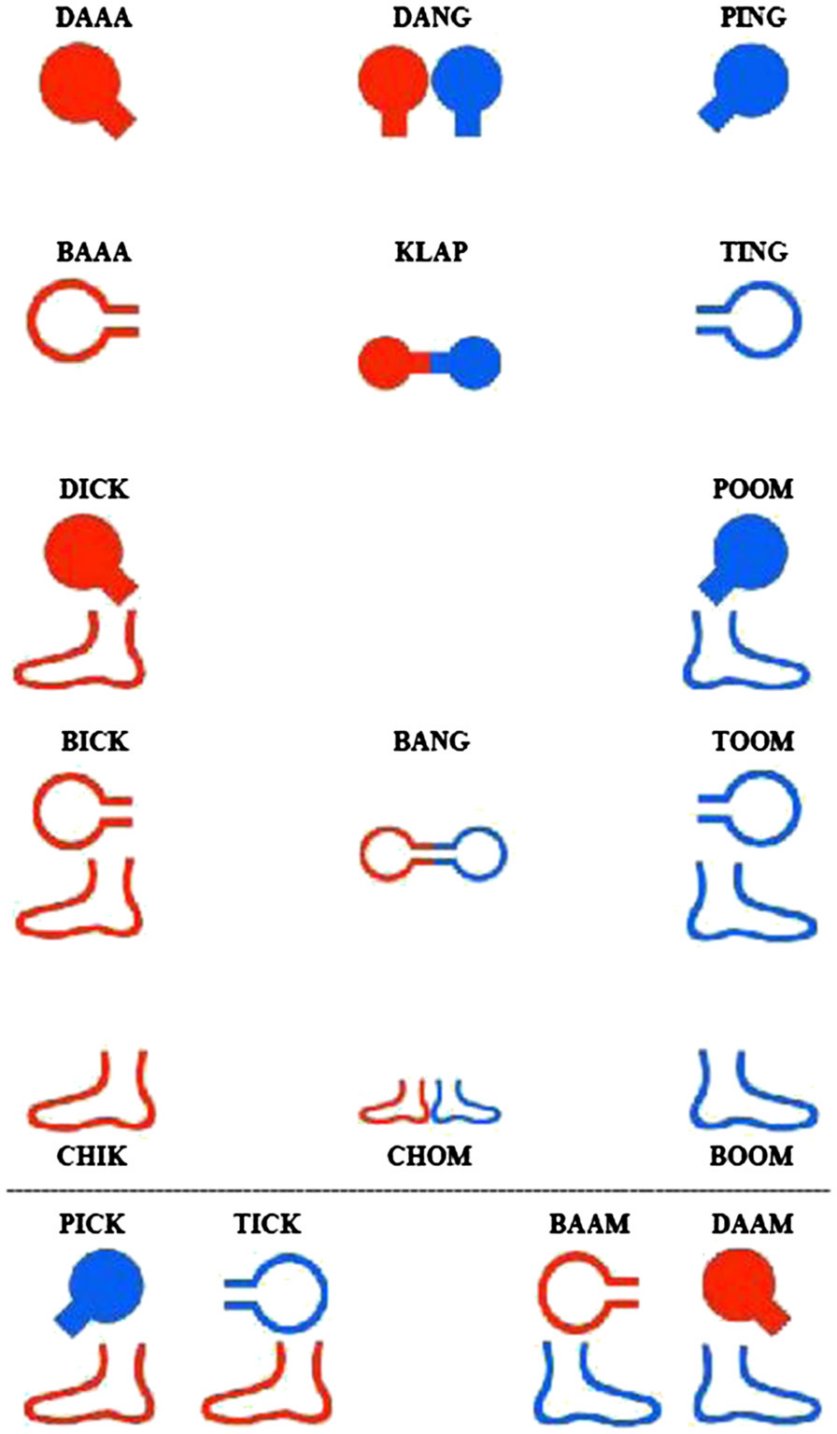
The RGRM note system with corresponding sound codes.

We use a method of multi-sensory stimulation of the brain which is based on rhythm and music and originally developed by jazz drummer Ronnie Gardiner. The therapy program, designed to help people with injuries and diseases of the central nervous system [73] is called Ronnie Gardiner Rhythm and Music method (RGRM™) and has since 1993 been implemented in health care and rehabilitation in Sweden. The method is based on the principle of neuroplasticity [34], and uses rhythm, music, colour, the voice, text, shapes and movement to stimulate coordination, balance, endurance, attention, memory, body image and social interactions. RGRM™ uses a unique note system (illustrated in Figure 2) that combines red and blue body symbols with corresponding sound codes and body movements. The symbols represent hands and feet and are displayed on a screen. The colours represent right and left brain activity, with the right brain (red) governing the left side of the body, and left brain (blue) governing the right side of the body. In total, there are 18 specific body movements and participants carry out the movements by clapping hands, tapping their hands on their knees and stamping their feet on the floor, without using any other tools than their own bodies.

The note system may be combined in a number of ways in order to stimulate different parts of the brain. A certified RGRM™ practitioner creates a piece of movement coordination (chorescore) and participants coordinate the series of movements while saying its name, all accompanied with rhythm and music. Through the stimulation of senses and the rhythmic activation of body movements and accompanying sound codes (derived from drum sounds), the right- and left-hand sides of the body are activated simultaneously, together with the stimulation of the left- and right brain hemispheres. The level of difficulty is adjusted with regard to the level of mobility and capabilities of the participants. At their individual pace the participants perform increasingly more complex sequences of rhythmic composite movements. If a participant cannot perform a certain movement the person is instructed to initiate/imagine the movement. The following parameters were selected as the intervention in the group T1 and T3: two sessions per week for twelve weeks, each session of 90 min in duration broken into 10 segments including a coffee break and concluded by a summary.

#### Therapeutic riding

TR is s multi-sensory treatment modality that combines physical activity with cognitive stimulation and emotional connection with the horse. Its goal is to improve posture, balance, coordination, muscular strength and cognitive functions, while offering an opportunity to improve motivation and self-esteem. The rhythmic and repetitive walking motion of a horse resembles the human walking gait, and the many textures, sounds and sights provide an enriched environment. We selected the following parameters as the intervention in the group T2: two sessions per week for twelve weeks, each session of 240 minutes in duration consisting of two segments, interaction with the horse prior to or after riding, and the riding itself. The treatment also includes preparation of the horse (grooming and equipping the horse with shabrack, voltage girth and bridle before the riding session, or their removal afterwards). The sessions are concluded by eating lunch or having refreshments together with the therapists and assisting personnel.

The TR is performed at a riding centre adapted for disabled and the riding sessions are held mostly outside in the paddock and inside during bad weather conditions. The sessions take place in groups of two to six participants who ride in pairs for 30 minutes, while the others are bystanders. The treatment plan as well as appropriate horses, equipment and exercises are selected in order to facilitate treatment goals, and to provide the most effective treatment for each patient along the intervention period. The sessions are led by a physiotherapist and an occupational therapist who both have strong equine background, as well as knowledge of disabilities related to stroke and education in TR.

Two assistants prepare the horses and lead them during the riding sessions and assist during the mounting on a ramp and the dismounting on the ground. During riding the participant sits on a shabrack (thick soft cover) and as a safety precaution, one assistant walks alongside the horse and another assistant leads the horse. Each riding session begins and ends with *relaxation and body awareness exercises* – deep, slow breathing, focusing and relaxing body parts starting out from shoulders towards feet, partly with the eyes shut while instructed to feel the horse’s movements through their own body. The mounted exercises are individually tailored to the subjects’ physical needs and ability to ride and include the following: 1. *Balance exercises*: maintaining balance while holding one or both arms sideways; putting the hand/hands on the head; riding in diagonals, circles, over low poles and weaving through cones; 2. *Trunk rotation*: reaching for the horse’s tail; holding a stick with both hands with elbows at the waist and then rotating the trunk to the sides; 3. *Exercises designed to train participants’ affected body parts*: simulating bicycling with the legs; reaching for the horse’s ears; lying prone with the arms around the horse’s neck and then rising again; grasping a tennis ball from the instructor in different directions; controlling the horse by holding the reins; 4. *A cognitive component*: taking part in the planning of the participant’s own riding in different directions and exercises; paying attention to the other equipage in the paddock while riding; following multiple oral instructions. Whenever the horse moves or when changes in speed or direction take place, postural adjustment is required. All exercises are, if possible, performed while the horse is moving and mostly the subjects ride at a walking pace although some participants try a few laps of trot.

### Evaluation procedure

At baseline, group equality will be determined regarding all descriptive and baseline variables.

The effects of the interventions are evaluated using a pre- and post-test design. The baseline evaluation phase precedes randomization and takes place 1−3 weeks prior to the first intervention session. The post-intervention evaluation is undertaken within 1−3 weeks and approximately six month after intervention completion, comprising the whole measurement battery. At three months after completion of the intervention phase, all study questionnaires are completed and blood samples are collected. A research coordinator, a nurse, a physiotherapist and a neuropsychologist perform the evaluation of participants throughout the trial. At all evaluation phases, measurements are being carried out in the same sequence throughout the trial. All questionnaires are posted to the participants beforehand, in order to have them completed prior to the appointment with the research coordinator. In order to ensure that the completion of the questionnaire has been correctly performed, the research coordinator goes through the questionnaire together with the participants by face-to-face interviews. The individual interviews are performed within four weeks after end of treatment and the focus groups are conducted six months after completion of the intervention by two experienced persons (occupational therapist and speech therapist), the latter being responsible for interviewing aphasic participants.

#### Measures of participant entry characteristics

The participant characteristics recorded are: gender, age, time since stroke onset both at entry to the trial and at each evaluative period (days), the type and site of the stroke lesion, previous stroke insult(s) in the same hemisphere, level of educational attainment and handedness. The Modified Rankin Scale (MRS) is used to describe the degree of disability or dependence in daily activities among the participants [74]. To describe the level of dependence/independence in personal and instrumental activities of daily living among the participants, the *ADL Staircase* is used [75], [76]. *The National Institutes Health Stroke Scales (NHISS)* are used to describe neurological deficit among the participants [75,77].

#### Outcome measures

**Figure 3:**
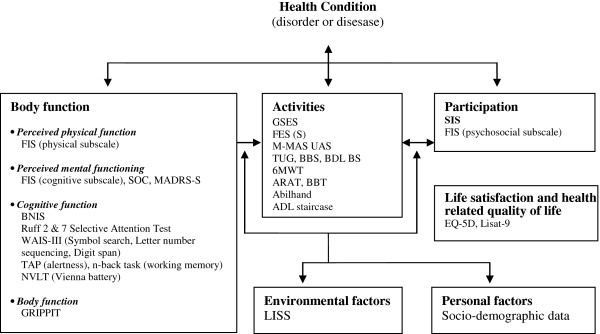
**International classification of functioning, disability and health (ICF) categorization of measures used in the present trial (for abbreviations see Table**2**).**

Reliable and valid measures are used for the outcome assessment and are classified according to the different domains of the ICF [66,78,79]. The ICF definitions are presented in Table 1. The outcome measures describe overall health status and functioning at six different perspectives: 1. *Body functions*: including the following subcategories: perceived physical and mental functioning and cognitive function; 2. *Activities;* 3. *Participation*; 4. *Environmental factors;* 5. *Personal factors* and 6. *Life satisfaction and quality of life* (not part of the ICF). The outcome measures used within each domain are presented in Figure 3, and the outcome measure abbreviations are listed in Table 2. For the below listed outcome measures sum scores are calculated when appropriate.

**Table 1:** ICF definitions

**Terminology**	**Definition**
Body functions/structure	Physiological functions of body systems including psychological. Structures are anatomical parts or regions of their bodies and their components. Impairments are problems in body function or structure.
Activity	The execution of a task by an individual. Limitations in activity are defined as difficulties an individual might experience in completing a given activity.
Participation	Involvement of an individual in a life situation. Restrictions to participation describe difficulties experienced by the individual in a life situation or role.

**Table 2:** Outcome measure abbreviations

**Abbreviation**	**Outcome measure**
ADL	Activities of Daily Living
ARAT	Action Reach Arm Test
BBS	Bergs Balance Test
BBL BS	Bäckstrand, Dahlberg, and Liljenäs Balance Scale
BBT	Box and Blocks Test
BNIS	Barrow Neurological Institute Screen for Higher Cerebral Functions
EQ-5D	EuroQol 5D
FIS	Fatigue Impact Scale
FES	Falls-Efficacy Scale
GSES	General Self-Efficacy Scale
LISS	Life Situation among spouses after the Stroke event questionnaire
Lisat-9	Life Satisfaction Checklist-9
MADRS-S	Montgomery-Åsberg Depression Rating Scale – Self rate
M-MAS UAS	Modified Motor Assessment Scale according to the Uppsala University hospital
NVLT	Non-Verbal Learning Test
Ruff 2 & 7 SAT	Ruff 2 & 7 Selective Attention Test
SIS	Stroke Impact Scale
SOC	Sense of Coherence
TAP	Test for Attentional Performance
TUG	Timed Up and Go
VAS	Visual Analogue Scale
WAIS	Wechsler Adult Intelligence Scale
6MWT	6 Minutes Walk Test

### Primary outcome measure

#### Participation

The main primary outcome is the stroke-specific, self-report, health status measure *Stroke Impact Scale (SIS, version 2.0)* which assesses several dimensions of health related quality of life: emotion, communication, memory and thinking, and social role function. The SIS also includes a question to assess the individual’s global perception of degree of recovery from stroke, with 0 indicating “no recovery” and 100 indicating “full recovery” and is used both in clinical and research settings [80]. In the present study, any increase equivalent to 10% of the total range of the scale is considered as an improvement. For the participation domain, the *psychosocial subscale* (20 items) of the *Fatigue Impact Scale* (FIS) is used [81].

### Secondary outcome measures

#### Body function and structure

As a measure of perceived physical functioning, the physical subscale (10 items) of the FIS [80] and the *Montgomery-Åsberg Depression Rating Scale* – self rate (MADRS-S) are used [82,83]. As a measure of perceived mental functioning, the *cognitive subscale* (10 items) of the FIS is used [81]. The participants’ ability to cope with stress is assessed using the 13-item version of The *Sense of Coherence* (SOC) scale [84]. Assessment of general cognitive level is done using the *BNI Screen for Higher Cerebral Functions* (BNIS) [85]. Psychomotor speed and selective attention are assessed using the *Ruff 2 & 7 Selective Attention Test*[86]. Psychomotor processing speed is also assessed by the *Symbol search*, a subtest from the Wechsler Adult Intelligence Scale (WAIS-III) [87]. Working memory is assessed using the *Letter-Number Sequencing* (LNS) in Wechsler Adult Intelligence Scale (WAIS-III) [88] and by a modified shorter version of the subtest *Digit span* in (WAIS-III). Alertness, simple psychomotor speed and working memory is assessed using the computerized *Test for Attentional Performance (TAP)*[89]. Non-verbal learning of visual patterns is evaluated by the *Non-verbal Learning Test* (NVLT) included in the computerized Vienna Test System [90]. After the performance of the above listed cognitive tests, the participants rate their experienced mental and physical fatigue using a *Visual Analogue Scale* (VAS) [91]. Grip strength is measured using a *Grippit*® instrument (AB Detektor, Göteborg, Sweden) [92].

#### Activity

Self-belief to cope with a variety of difficult demands in life is assessed using the *General Self-Efficacy Scale* (GSES) [93]. As a measure of perceived physical functioning, the *physical subscale* of the FIS (10 items) is used [80]. Perceived confidence in task performance is measured using Swedish modification of the *Falls-Efficacy Scale –* FES (S) [94]. Balance is evaluated by the *Berg Balance Scale* (BBS) [95]. Even though there are no formal validity or reliability data published yet, the Swedish clinical observer-assessed *Bäckstrand, Dahlberg, and Liljenäs Balance Scale* (BDL BS) is used as a complement to BBS in order to detect changes in individuals with light to moderate balance disturbances [96,97]. The BDL BS is translated into English [98]. Motor recovery is assessed using the *Modified Motor Assessment Scale according to the Uppsala University hospital* (M-MAS UAS) [99]. Walking capacity is measured using the *6-minute walk test* (6MWT) [100]. Mobility is measured by the *Timed “up and Go”* (TUG) test [101]. Upper limb function is determined using the *Action Research Arm Test* (ARAT) [102]. Manual dexterity is measured using the *Box and Block Test* (BBT) [103] and manual ability is assessed using the *Abilhand* questionnaire [104]. The level of dependence/independence in personal and instrumental activities of daily living among the participants is evaluated by the *Activities of Daily Living* (*ADL) staircase*[75].

#### Environmental factors

The life situation of spouses is evaluated by the *Life Situation among Spouses after the stroke event questionnaire* (LISS) [105].

#### Life satisfaction and health related quality of life

Life satisfaction is measured using the *Life Satisfaction Checklist* - LiSat-9 [106]. Health-related quality of life is measured using the *EuroQol* (EQ-5D) [107,108].

#### Qualitative outcome measures

Both individual interview and focus groups methodology are used. Selected participants from each intervention group are interviewed when treatment is finalized in order to study their experiences from the two interventions and how they impact on their life situation. As a complement to individual interviews, focus groups are conducted six months after end of treatment to facilitate the opportunity for selected participants to interact during a group discussion based on experiences from the interventions and the subsequent impact on life satisfaction and performance in daily life. Both interviewers are present at the focus groups in which the occupational therapist has the main responsibility for probing questions using a semi-structured interview guide. The speech therapist assists aphasic participants in the discussion.

The participants’ expectation of the treatment is measured using a self-constructed questionnaire containing the following two questions; 1. How effective do you believe the treatment is for individuals with a history of stroke? with four possible answers ranging from “very effective” to “not effective”, and; 2. What impact do you believe the treatment has on the difficulties you have following your stroke? with four possible answers ranging from “huge impact” to “no impact”.

### Blood sampling and analysis

Blood samples are taken for the analysis of biomarkers*.* The participants come fasting and the blood is drawn between 8.00 and 10.00 a.m. At all four evaluation periods blood samples are taken from each participant and serum and EDTA plasma are prepared and stored in aliquots at -70°C for use for biochemical analysis, proteomic and quantitative real time PCR. Further, buffy coat obtained from EDTA blood is aliquoted and either frozen directly or frozen in a cryoprotectant at -140°C in order to get individual cells intact for quantitative single cell rtPCR. In addition, blood drawn into a PAXgene tube will serve for whole blood preparation for mRNA preparation and gene expression profiling. After being in room temperature for at least 4 hours these samples are frozen at -20°C for 24 hours and then at -80°C. Depending on the clinical outcome, mRNA will be prepared from samples from selected participants.

Protein presence is detected and the relative quantity of individual proteins in blood plasma is measured using relative quantification with mass spectrometry and isobaric tagging reagents that yield amine derived peptides for quantification [109,110]. The quantitative proteomics analysis will provide information about relative protein ratios in selected patients before and after intervention. This explorative part of the study is aimed to form a basis for later validation of possible candidate biomarkers.

### Current status

A total of 84 participants were randomised and have completed the intervention and the recruitment is expected to be completed by August 2013. The drop-out rate in the study has been 7%, including one participant allocated to T3 who suffered a new stroke, and another participant randomized to group T3, who deceased during the treatment period. Two of the drop-outs were randomized to group T3 and one was allocated to group T2. Recruitment proceeds and follow-up is on-going, trial results are expected in 2014.

## Discussion

Stroke represents one of the most costly and long-term disabling conditions in adulthood worldwide and there is a need to determine the effectiveness of rehabilitation programs in the late phase after stroke for which currently only limited scientific support exists. The general belief has been that treatment of individuals in the late phase of stroke is of no benefit. Today, the concept of brain plasticity gives hope for improvements in rehabilitation that go beyond spontaneous recovery of function [111]. The rehabilitation process should encompass all dimensions of a stroke survivor’s life, and rehabilitation programs that address both the social and physical needs of the patients, preferably individually tailored, are therefore highly desirable.

The present study was structured and designed according to CONSORT guidelines [112], in order to enable its reproduction in both clinical and research settings. The rationale for the study derives from the fact that various forms of enriched environments and multimodal stimulation components have positive influence on motivation and psychosocial well-being and facilitate multiple processes in the brain, leading to structural regeneration and functional recovery [23-33].

The results of the trial may contribute to the knowledge about the effects of rhythm and music-based therapy and TR in late stage post-stroke rehabilitation on overall health status and functioning, and might also help to identify the predictive parameters of therapeutic success. We anticipate that the results will have important implications for health care policy by increasing the scientific basis for such interventions and contribute to the implementation of effective rehabilitation programs in the clinical praxis.

## Abbreviations

ADL: Activities of Daily Living; ARAT: Action Reach Arm Test; BBS: Bergs Balance Scale; BBL BS: Bäckstrand Dahlberg and Liljenäs Balance Scale; BBT: Box and Blocks Test; BNIS: Barrow Neurological Institute Screen for higher cerebral functions; EQ-5D: EuroQol 5D; FIS: Fatigue Impact Scale; FES: Falls-Efficacy Scale; GSES: General Self-Efficacy Scale; LISS: Life Situation among Spouses after the Stroke event questionnaire; Lisat-9: Life Satisfaction checklist-9; LOCF: Last observation carried forward; MADRS-S: Montgomery-Asberg Depression Rating Scale – self rate; MID: Minimal Important Difference; M-MAS UAS: Modified Motor Assessment Scale according to the Uppsala Academy hospital; MRS: Modified Rankin Scale; NHISS: The National Institutes Health Stroke Scales; NMT: Neurologic Music Therapy; NVLT: Non-Verbal Learning Test; Ruff 2 & 7 SAT: Ruff 2 & 7 Selective Attention Test; SIS: Stroke Impact Scale; SOC: Sense of Coherence; TAP: Test for Attentional Performance; TUG: Timed Up and Go; VAS: Visual Analogue Scale; WAIS: Wechsler Adult Intelligence Scale; 6MWT: 6 Minutes Walk Test; ICF: International Classification of Functioning, disability, and health; RGRM: Ronnie Gardiner Rhythm Music method; TR: Therapeutic riding.

## Competing interests

The authors declare that they have no competing interests.

## Authors’ contributions

All authors participated in the conception, planning and design of the study. MN, MP, MAP, CB and LBK have contributed to the financing of the study. The project leader LBK has coordinated the planning of the trial and is responsible for day-to-day management of the study and its implementation, primarily supported by CB and ÅLN. MP, MN and MAP have also been responsible for the biomarker part of the study. All authors contributed to writing of the current manuscript and all have approved the final version.

## Pre-publication history

The pre-publication history for this paper can be accessed here:

http://www.biomedcentral.com/1471-2377/12/141/prepub
